# Data on the mRNA expression by in situ hybridization of Wnt signaling pathway members in the mouse uterus

**DOI:** 10.1016/j.dib.2017.03.047

**Published:** 2017-04-08

**Authors:** Jyoti Goad, Yi-An Ko, Shafiq M. Syed, Yazmin J. Crossingham, Pradeep S. Tanwar

**Affiliations:** School of Biomedical Sciences and Pharmacy, University of Newcastle, Callaghan, New South Wales 2308, Australia

**Keywords:** Wnt, β-catenin, Uterus, Endometrium, Glands

## Abstract

Wnt signaling plays an important role in uterine organogenesis and oncogenesis. Our mRNA expression data documents the expression of various Wnt pathway members during the key stages of uterine epithelial gland development. Our data illustrates the expression of Wnt signaling inhibitors (Axin2, Sfrp2, Sfrp4, Dkk1 and Dkk3) in mice uteri at postnatal day 6 (PND 6) and day 15 (PND 15). They also describe the expression pattern of the Wnt ligands (Wnt1, Wnt2, Wnt2b, Wnt3, Wnt3a, Wnt5b, Wnt7b, Wnt8a, Wnt8b, Wnt9a, Wnt9b, Wnt10a and Wnt10b) in mice uteri with or without progesterone treatment. Detailed interpretation and discussion of these data is presented in the research article entitled “Differential Wnt signaling activity limits epithelial gland development to the anti-mesometrial side of the mouse uterus” [1].

**Specifications Table**

**Table:** 

Subject area	*Biology*
More specific subject area	*Wnt signaling /uterine epithelial gland development*
Type of data	*Figures*
How data was acquired	*in situ hybridization and microscopy*
Data format	*Analyzed*
Experimental factors	*The uteri were collected from time mated females or mice exposed to progesterone at specific time points and were fixed in 10% neutral buffered formalin overnight at room temperature. The fixed tissues were paraffin embedded and sectioned at* 4 µm *thickness. These sections were deparaffinised and exposed to probes targeting the different Wnt pathway members.*
Experimental features	*Assessment of mRNA expression of the Wnt pathway members in endometrial epithelium and stromal cells of normal and progesterone treated mice uteri.*
Data source location	*N/A*
Data accessibility	*All the relevant data are supplied in this article*
Related research article	*“Differential Wnt signalling activity limits epithelial gland development to the anti-mesometrial side of the mouse uterus”**[1]*.

**Value of the data**

• The data presented provide useful insight into the expression pattern of Wnt inhibitors and ligands in the mouse uterus during the process of gland development.
• These data provide evidence that Wnt signaling antagonists are expressed in both uterine epithelium and stroma.
• However, the expression of Wnt1, Wnt2, Wnt2b, Wnt3, Wnt3a, Wnt5b, Wnt7b, Wnt8a, Wnt8b, Wnt9a, Wnt9b, Wnt10a and Wnt10b is absent in mice uteri.
• Progesterone treatment in the neonatal mice, does not affect the expression of Wnt1, Wnt2, Wnt2b, Wnt3, Wnt3a, Wnt5b, Wnt7b, Wnt8a, Wnt8b, Wnt9a, Wnt9b, Wnt10a and Wnt10b.
• These data are useful for the scientific community members interested in examining the role of Wnt signaling in female reproductive tract biology and carcinogenesis.

## 1 Data

**Fig. 1:**
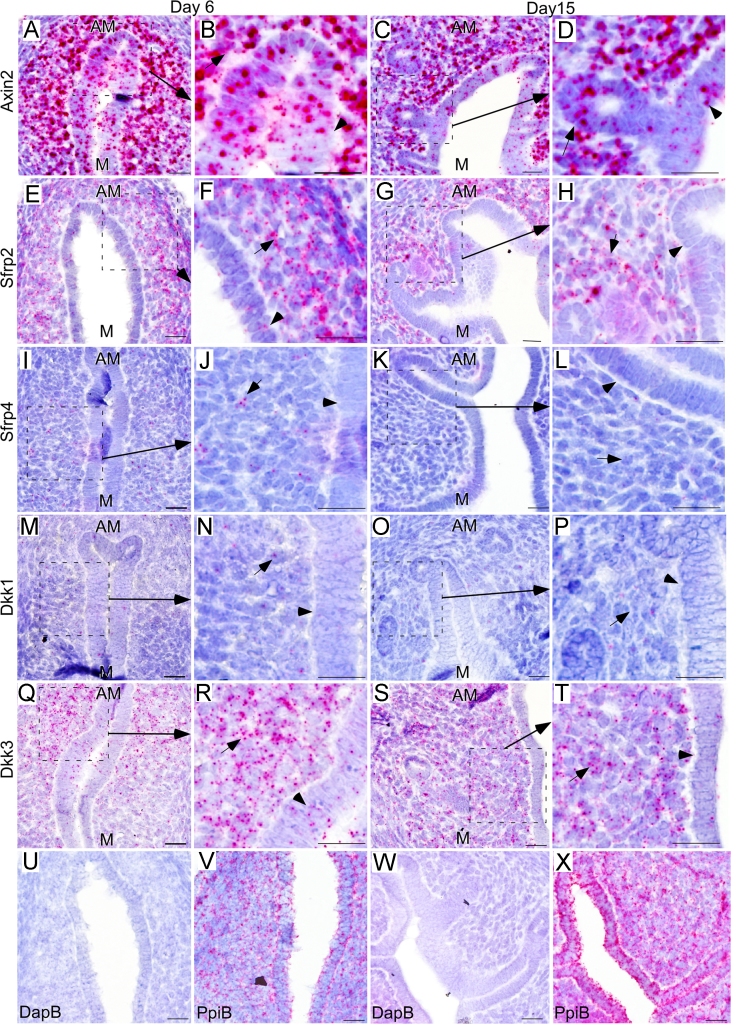
Expression pattern of Axin2 (A-D), Sfrp2 (E-H), Sfrp4 (I-L), Dkk1 (M-P), Dkk3 (Q-T) at PND 6 and PND15 in mice uteri (*N*=3/each). The bacterial gene, dihydrodipicolinate reductase (DapB), and the housekeeping gene, peptidylprolyl isomerase B (Ppib), were used as negative and positive controls, respectively (U-X). The luminal epithelium is marked by black arrowheads, and uterine glands by black arrows. AM: anti-mesometrial, M: mesometrial. Bar: 20 µm.

**Fig. 2:**
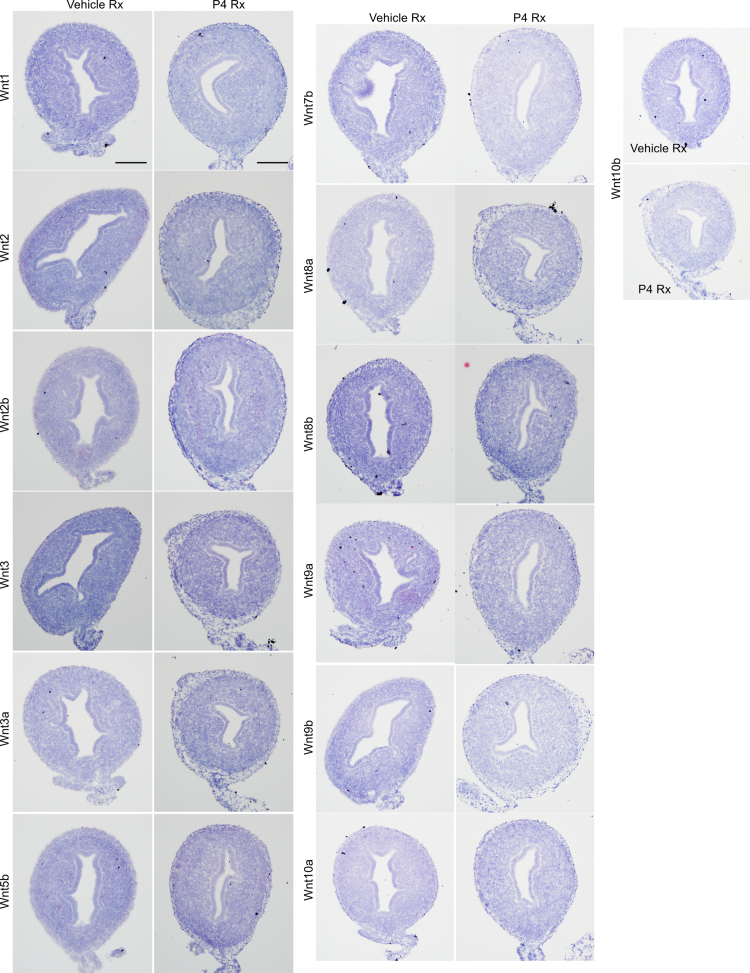
Wnt1, Wnt2, Wnt2b, Wnt3, Wnt3a, Wnt5b, Wnt7b, Wnt8a, Wnt8b, Wnt9a, Wnt9b, Wnt10a, and Wnt10b expression in progesterone or vehicle treated uteri. *N*=3/each. Bar: 100 µm.

Fig. 1 showed that Axin2, Sfrp2, Sfrp4, Dkk1 and Dkk3 mRNA expression is present in the uterine epithelium and/or stromal cells. Progesterone treatment of neonatal mice suppresses endometrial gland development but has no effect on the expression of Wnt ligands: Wnt1, Wnt2, Wnt2b, Wnt3, Wnt3a, Wnt5b, Wnt7b, Wnt8a, Wnt8b, Wnt9a, Wnt9b, Wnt10a and Wnt10b (Fig. 2).

## 2 Experimental design, materials and methods

### 2.1 Animals

All the experimental procedures were approved by the Animal care and ethics committee, the University of Newcastle, Australia. For animal care and experimental procedures guidelines of: the New South Wales Animal Research Act, New South Wales Animal Research Regulation, and the Australian code for the care and use of animals for scientific purposes, were followed. Mice were housed under standard housing conditions and maintained on C56BL/6;129SvEv mixed genetic background. Mice were time mated and uterine tissue samples were collected at PND 6 and 15 (*N*=3/each).

### 2.2 Progesterone treatment

TCF-GFP mice were treated with progesterone (Sigma) as described in [2]. Briefly, mice were subcutaneously injected with progesterone (50 μg/g) or vehicle daily from postnatal day 3 to postnatal day 11. At postnatal day 11, uteri were collected and fixed in 10% neutral buffered formalin overnight at room temperature and paraffin embedded.

### 2.3 in situ hybridization and microscopy

Tissue sections from uteri of three different mice per group were deparaffinized with xylene and rehydrated in a series of graded ethanol. These sections were air dried on absorbent paper with the tissue section face-up to remove excess ethanol. These sections were then exposed to RNAscope® hydrogen peroxide for approximately 10 min, followed by antigen retrieval for 10 min (RNAscope® Target Retrieval Reagents). in situ hybridization was performed using RNAscope® 2.5 HD Assay-RED kit (Advanced Cell Diagnostics, Hayward, CA) [3]. The RNAscope probes used in this study were - DapB (EF191515), PPIB (NM_011149.2), Wnt1(NM_021279.4), Wnt2 (NM_023653.5), Wnt2b (NM_009520.3), Wnt3 (NM_009521.1), Wnt3a (NM_009522.2), Wnt5b (NM_001271757.1), Wnt7b (NM_009528.3), Wnt8a (NM_009290.2), Wnt8b (NM_011720.3), Wnt9a (NM_139298.2), Wnt9b (NM_011719.4), Wnt10a (NM_009518.2), Wnt10b (NM_011718.2), Axin2 (NM_015732.4), Sfrp2 (NM_009144.2), Sfrp4 (NM_016687.3), Dkk1 (NM_010051.3) and Dkk3 (NM_015814.2). Images were taken using Olympus DP72 microscope.
