# Supplementary material for: Data on the mRNA expression by in situ hybridization of Wnt signaling pathway members in the mouse uterus
Source: Data Brief. 2017 Apr 8;12:208–12. doi: 10.1016/j.dib.2017.03.047 (PMC5393313; doi:10.1016/j.dib.2017.03.047)
Supplement: Supplementary file 1 — Supplementary material [file mmc1.pdf]

We wish to confirm that there are no known conflicts of interest associated with this publication and there has been no significant financial support for this work that could have influenced its outcome.

Regards,

Pradeep Tanwar
ARC Future Fellow
Cancer Institute NSW CD Fellow
Senior Lecturer, University of Newcastle
Callaghan 2308 NSW Australia
02-49215148
pradeep.tanwar@newcastle.edu.au
